# A Deep Learning Approach for Biped Robot Locomotion Interface Using a Single Inertial Sensor

**DOI:** 10.3390/s23249841

**Published:** 2023-12-15

**Authors:** Tsige Tadesse Alemayoh, Jae Hoon Lee, Shingo Okamoto

**Affiliations:** Department of Mechanical Engineering, Graduate School of Science and Engineering, Ehime University, Bunkyo-cho 3, Matsuyama 790-8577, Ehime, Japan

**Keywords:** motion synthesis, deep learning, walking controller, inertial sensor

## Abstract

In this study, we introduce a novel framework that combines human motion parameterization from a single inertial sensor, motion synthesis from these parameters, and biped robot motion control using the synthesized motion. This framework applies advanced deep learning methods to data obtained from an IMU attached to a human subject’s pelvis. This minimalistic sensor setup simplifies the data collection process, overcoming price and complexity challenges related to multi-sensor systems. We employed a Bi-LSTM encoder to estimate key human motion parameters: walking velocity and gait phase from the IMU sensor. This step is followed by a feedforward motion generator-decoder network that accurately produces lower limb joint angles and displacement corresponding to these parameters. Additionally, our method also introduces a Fourier series-based approach to generate these key motion parameters solely from user commands, specifically walking speed and gait period. Hence, the decoder can receive inputs either from the encoder or directly from the Fourier series parameter generator. The output of the decoder network is then utilized as a reference motion for the walking control of a biped robot, employing a constraint-consistent inverse dynamics control algorithm. This framework facilitates biped robot motion planning based on data from either a single inertial sensor or two user commands. The proposed method was validated through robot simulations in the MuJoco physics engine environment. The motion controller achieved an error of ≤5° in tracking the joint angles demonstrating the effectiveness of the proposed framework. This was accomplished using minimal sensor data or few user commands, marking a promising foundation for robotic control and human–robot interaction.

## 1. Introduction

Humanoid robots have been garnering increasing interest in society due to advancements in both hardware and software, along with heightened expectations for their application in areas such as disaster response [1] and human interaction [2,3]. This human-like ability makes them suitable to execute tasks in environments designed for humans. Additionally, another innovative use of these human-like abilities is in the field of assistive devices such as exoskeleton robots, leveraging their sensory capabilities and active mobility [4]. Not only for optimal control but also for the proper design of humanoid and assistive robots, human motion data are indispensable [5,6]. Human motion data are the sole source of information that gives human traits to different mechanical structures as well as digital human characters. For these varied applications, it is crucial for robots to replicate human motion with high fidelity. However, this task is challenging due to the inherent differences in body structure and mechanical properties between humanoid robots and humans.

The primary challenge lies in establishing a link between the states of the robot and human movements, a process often referred to as motion retargeting. For certain robots with less mechanical complexity, like manipulators, the link can be relatively straightforward. However, for robots with more structural complexity, such as bipeds and quadrupeds, establishing the correspondence is far from straightforward due to the inherent ambiguity in the problem. Hence, various model-driven and data-driven retargeting or tracking controllers have been developed in recent years. Model-based control methods rely on the mathematical model of the mechanical structure to design an optimal tracking controller. Due to the inherent complexity of mathematical models of robots, this control strategy is mainly adopted in more simplified robotic mechanisms [7,8,9,10,11,12]. On the other hand, data-driven approaches are suitable for multilegged robots with different morphology [13,14].

Model-driven methods have been the conventional methods in this retargeting human motion field. A study by [7] proposed an inverse kinematics formulation that enables control of end-effectors’ position and orientation of the biped robot, as well as the robot’s center of mass given data prepared from physics engine simulators. A study by [8] introduced dynamic filters in the retargeting process of the whole-body data collected from 17 inertial sensors to prevent motions that could cause the humanoid robot to fall. Other studies by [9,12] solved an optimization problem and used inverse kinematics strategies to track human-motion-capture data using biped robots. Ref. [12] developed a whole-body teleoperation architecture that enables intuitive and natural interaction between the human operator and the humanoid robot at the configuration space level, whereas [9] divided the retargeting problem into morphing the human model to the robot model, motion planning for the robot, and then inverse kinematics of human-motion-capture data. Unlike the previously mentioned studies, ref. [11] developed a differentiable optimal control (DOC) framework that interfaces with various sources of motion data and allows for the retargeting of motions from animals or animations onto quadruped robots with significantly different proportions and mass distributions. The DOC framework is utilized to optimize parameters within a set of retargeting objectives, accounting for variations in proportions and degrees of freedom between the motion data source (e.g., animals, animations) and the quadruped robot. A study by [15,16] introduced a control framework tailored for humanoid robots, enabling them to simultaneously track motion capture data while maintaining balance. Ref. [15]’s proposed controller consists of balance and tracking controllers. While a linear quadratic regulator computes the desired input necessary for maintaining balance, the tracking controller computes the joint torques that minimize the deviation from the desired inputs, ensuring the motion capture data are tracked while considering the full-body dynamics. Meanwhile, ref. [16] proposed a Cartesian control approach where a set of control points on the humanoid are selected. The robot is then virtually connected to those points via translational springs whose forces drive a simplified simulation of the robot dynamics.

The data-driven approaches, on the other hand, are dominated by neural-network-based retargeting methodologies. A study by [13] introduced CrossLoco, a guided unsupervised reinforcement learning framework that learns robot skills and their correspondence to human motions simultaneously. The core is a cycle-consistency-based reward term designed to maximize mutual information between human motions and robot states. This approach includes both robot-to-human and human-to-robot reconstruction networks. Another significant contribution in the field of animation synthesis and editing is based on a deep learning framework that can automatically learn an embedding of motion data in a non-linear manifold using a vast set of human motion data [17]. This model allows for the synthesis of novel motion via interpolation and offers users the ability to produce realistic human motion sequences from intuitive inputs. Another study by [14] introduced a reinforcement-learning-based approach to train a real-world bipedal robot to replicate movements derived directly from human motion capture data. They claimed to have achieved a smooth transition from simulated to real-world robot execution, eliminating the need for real-world training iterations or offline procedures. Human motion data are also used in exoskeleton robot control methods. A study by [4] presented an adaptive radial basis function (RBF) network and feedforward control scheme for lower limb exoskeleton robots based on human gait data. The study aimed to make the exoskeleton move similarly to humans. The adaptive RBF network and feedforward controllers compensate for uncertain model parameters and the input torque of the robot. They claimed that their results ensured effective assistant rehabilitation training.

All the above-mentioned studies mainly focus on directly reconstructing the human motion data on an animation character or robot without room for user interaction and customization of the output. Thus, it is difficult to synthesize a new set of motions for the robot. Generating new sets of motion from existing motion data without the need to collect data again is important as all sets of motion cannot be covered during data collection. To address this issue, studies by [18,19] introduced a phase-functioned neural network and a mode-adaptive neural network, which are trained on a vast human and dog dataset, to synthesize high-quality motions that adapt to various geometric environments in real time for a biped and quadruped animation characters. Trained models were able to create new sets of motions from user inputs, such as direction and pace. These studies highlight the potential of neural networks in capturing and synthesizing human and animal motions, paving the way for more realistic and responsive animations. However, these efforts predominantly rely on kinematics-based simulations, where state information is directly transferred into the character space without considering the underlying dynamics. Such a direct transfer, while effective in replicating the visual semblance of motion, often lacks the depth and realism of human biomechanics.

In most of the studies, the primary emphasis has been on retargeting given human motion data via simulated or actual robotic systems, with a notable absence of capabilities to generate new motion types. For locomotive robots, the ability to produce and subsequently track new motion types gives the system dynamic versatility, enabling a diverse range of motion activities [18,19]. Refs. [18,19] proved that having only a few independent high-level user commands is enough to generate a completely new motion set from an animal-motion-trained network. However, unlike [18,19], such parameters can be computed from only a few wearable sensors without the need for industry-level motion capture systems [20,21,22]. These studies implemented estimation gait parameters (such as gait speed and specific gait events) utilizing deep learning methods from a few inertial sensors (≤3).

In this study, human motion is retargeted to a biped robot using only a single inertial sensor fixed on the human pelvis. The entire framework comprises three parts: human motion parametrization, biped motion synthesis from high-level user commands, and inverse dynamics tracking controller for the generated motion. It is designed in a sequential encoder–decoder–controller configuration. Unlike autoencoders, both models are trained separately with human walking motion data. The encoder is first trained and outputs two high-level gait parameters namely the pelvis velocity and the gait phase. Since velocity is derived from the integration of acceleration, a neural network effective in handling long sequences and their ability to capture temporal dependencies is favorable [23,24]. Hence, a Bi-LSTM (bidirectional long short-term memory) network is used for the encoder network. The encoder outputs will then be used to train the decoder network, which is a set of fully connected layers. For the simplicity of the network, a fully connected network was chosen. The decoder then translates the input parameters into a motion represented by joint angles and pelvis displacement. To generate new sets of motion for the biped robot, the encoder will be replaced with a Fourier series parameter generator which accepts gait period and walking speed as input. The synthesized motion data are then fed to a constraint-consistent inverse dynamics robot controller as a reference motion, which employs a joint-space control algorithm to track and replicate the motion. It is worth noting that the encoder and decoder networks were trained using ground truth data sourced from an optical motion capture system and insole sensor data. Therefore, the contribution of this study is as follows:

- The design of a human–robot interface system from single inertial sensor data.
- The development of a deep learning algorithm to extract high-level walking motion gait parameters from a single pelvis inertial sensor. The parameters are pelvis velocity and gait phase.
- The design of a new methodology to synthesize completely new walking motions at different paces from user inputs.
- The development of a support consistent inverse dynamics control algorithm for a biped robot that tracks a generated reference motion.

The paper is organized as follows. After this introduction, which includes a brief discussion of the background and related works, the paper will explore the hardware and data collection methods. This is followed by an in-depth examination of the main motion synthesis process which will then lead to the results and discussion and, finally, the conclusion.

## 2. Hardware and Data Acquisition

**Figure 1:**
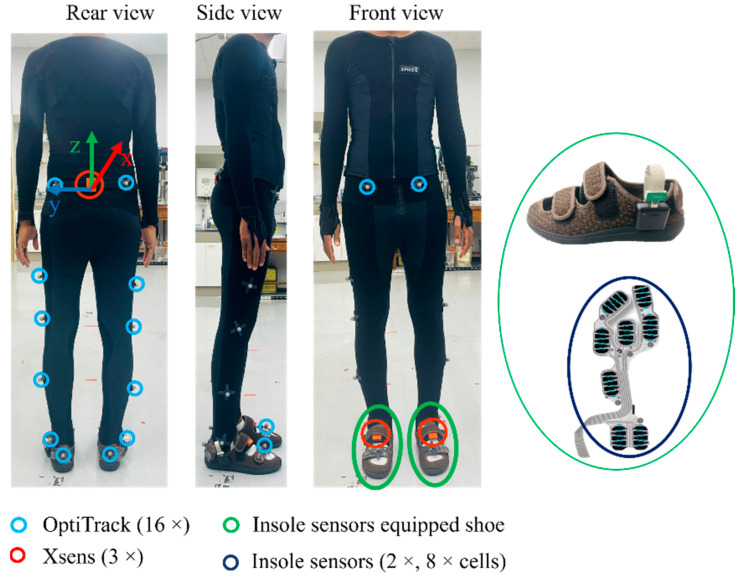
System experiment setup.

This section details the data collection process, the devices utilized, and the applied preprocessing methods of the study. Data measurement took place in a 6 × 3 m^2^ indoor space, where the subject was instructed to walk along the longer 6 m line. Data collection involved three motion sensing devices: optiTrack (an optical motion capture system), MTw Awinda (inertial motion sensor), and an insole pressure sensor as shown in Figure 1. The sensor systems are described in detail in the following subsections. Given the limited space, the subject walked along the 6 m line and stopped at its end, turned around, and paused briefly before walking back. The brief stops at each end of the 6 m line, accounting for nearly half of the collected data, were used to separate the walking data. In total, one hour of motion data from one subject was gathered. During the data collection, the subject was instructed to walk with varying stride lengths and speeds.

Inertial sensor: The IMU (inertial measurement unit) sensor used in this study, known as MTw Awinda, is manufactured by Movella Inc., headquartered in Henderson, NV, USA. Each MTw Awinda sensor, measuring 47 × 30 × 13 mm and weighing 16 g, communicates wirelessly at 2.4 GHz with an Awinda station connected to a PC via USB. The Awinda station guarantees time synchronization accuracy up to 10 μs. As shown in Figure 1, the subject wore three Xsens MTw Awinda sensors: one on the waist and two attached to the dorsum of both feet using non-slip Velcro tapes. The waist sensor was the primary one for motion generation, while the other two on the feet were attached solely for synchronization checking between the insole and the inertial sensors. The MTw Awinda sensors were accompanied by complimentary software named MT Manager 2019, capable of recording and exporting both raw inertial and orientation data of each sensor. For this study, only the three-axis accelerometer and three-axis gyroscope data were used. The motion data sampling rate was set to 100 Hz, aligning with the common sampling rate supported by all sensing systems. Since both the Awinda station and OptiHub of the motion capture support external syncing, data from both systems were synced using an intermediate BNC cable.

OptiTrack motion capture: This is an optical motion capture system that is regarded as the gold standard in motion measurement systems. The experimental space was equipped with eleven optiTrack Flex 13 motion capture cameras from NaturalPoint, located in Oregon, United States. The camera had a 1.3 MP resolution, 8.3 ms latency, +/− 0.20 mm 3D accuracy, and a frame rate of 120 FPS. After calibrating the cameras, the subject wore a special black suit with sixteen reflective markers attached to the lower body, as recommended by Motive 2.3.1.1 (the accompanying software). This optical motion capture system was accompanied by commercial software named Motive. Using this software, the pelvis position and lower limb joint angles were exported.

Insole pressure sensor: In addition to the inertial and motion capture sensors, the subject wore shoes fitted with a smart ActiSense Kit, manufactured by IEE, located in Luxembourg S.A. The kit comprised a smart insole and an electronic control unit (ECU) for recording motion data. The smart insole sensor, depicted in Figure 1, included eight pressure cells strategically placed in high foot-to-ground impact areas (heel left, heel right, arch, met 1, met 3 met 5, hallux, and toes). Similar to the other two systems, its sampling frequency was set at 100 Hz.

Data collection protocol: Initially, the selected healthy male subject wore a special black suit. The suit was specially designed to stretch and fit the body tightly, ensuring less skin artifact effect. Its outer surface had Velcro tape to firmly secure the reflective markers and the pelvis IMU sensor. First, calibration of the optical motion capture was performed inside the 6 × 3 m^2^ indoor space. Subsequently, the subject put on all three sensing devices. The subject then walked back and forth along the 6 m distance. To aid in the synchronization process, the subject stopped for two to three seconds and stamped at the start and end of each 6 m walk. The subject was instructed to walk at three different paces—slow, medium, and fast—and could switch between them at any time. However, the decision of how slow and how fast was left to the subject’s intuition. In addition, the subject was instructed to walk at different stride lengths.

Preparation: During the preparation stage, the synchronization of the three motion data modalities was verified and confirmed. As mentioned earlier, all three sensing systems recorded data at the same frequency of 100 Hz. Despite the uniform sampling frequency, slight misalignments could happen due to factors like clock drifts, data loss, buffering, and processing delays. Any misalignments were checked manually. The inertial sensor and the motion capture systems were already synced during data measurement. To synchronize the insole sensor data with the other two systems, the stamping events were used. As the foot–ground contact results in a sharp rise in pressure and a sharp decrease in acceleration, misalignments were manually corrected by checking the sharp changes in the inertial and insole sensors’ data. Furthermore, by identifying these distinct points in the motion data, turning motion data were excluded. Hence, only walking data were used for further processing in this study.

Finally, the gait cycle and heading axis were adjusted. Phases were used to represent the gait cycle. Each gait cycle was represented by a phase ranging from ‘0’ to ‘1’ with ‘0’ denoting the toe-off event of the right foot and ‘1’ denoting the next right foot toe-off (a stride). Since the subject changed their direction every 6 m during the back-and-forth motion, the heading direction was transformed so that it was always along the +*x*-axis.

## 3. Methods

**Figure 2:**
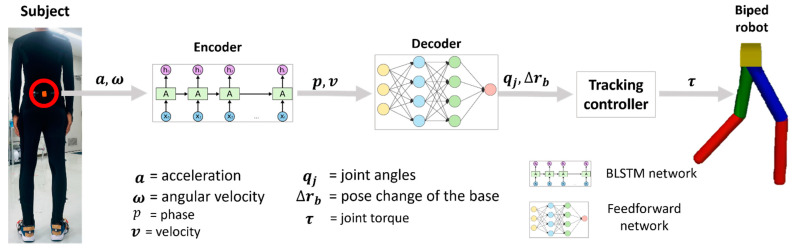
System overview of mode-I, where motion parameters v and p are computed from pelvis inertial data.

**Figure 3:**
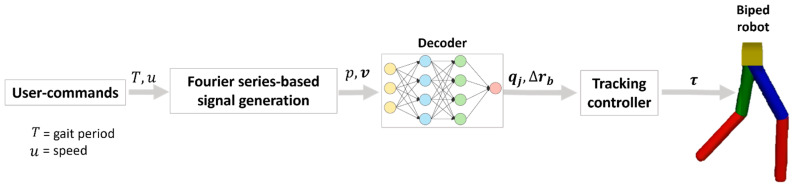
System overview of mode-II, where motion parameters v and p are computed from Fourier series signal generator.

In this section, the procedures of the proposed framework will be explained. The proposed framework consists of three sequential parts: motion parametrization (encoder), motion synthesis (decoder), and motion control (tracking controller). The framework was implemented using two modes, mode-I and mode-II, depending on the source of the input data. In mode-I, the input data originate directly from the human pelvis inertial sensor, whereas in mode-II, they come from user commands specifying walking speed and gait period. Each mode comprises different computational building blocks as illustrated in Figure 2 and Figure 3. In mode-I, the subject’s inertial data are fed directly into a Bi-LSTM encoder network, which then computes the subject’s walking velocity and phase. These signals are subsequently input into a feedforward decoder network, which outputs the leg joint angles and position changes of the base link. These quantities are then passed to a motion tracking controller which drives the biped robot. This study investigates four leg joints, specifically the extension/flexion of the hip and knee joints, as well as sagittal plane pelvis position changes. On the other hand, in mode-II, the encoder block is replaced by a Fourier series signal generator, which generates phase and velocity using high-level input parameters like gait period and walking speed.

### 3.1. Motion Parameterization

**Figure 4:**
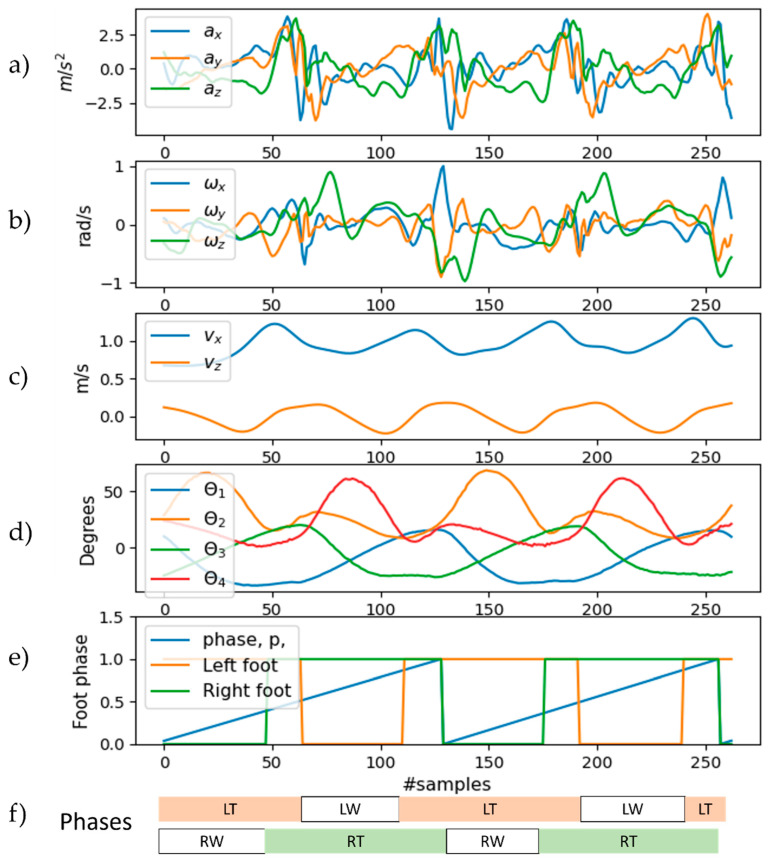
Example of human walking motion data. (**a**) Pelvis acceleration; (**b**) pelvis angular velocity; (**c**) pelvis velocity; (**d**) joint angle ($\theta_{1},\theta_{2},\theta_{3}$, and $\theta_{4}$ are the extension/flexion angles of the right hip, right knee, left hip, and left knee joint angles, respectively); (**e**) phase and foot state (LT = left stance; LW = left swing; RT = right stance; RW = right swing); (**f**) pictorial representation of the gait phase.

Before discussing the details, a brief introduction of the collected motion data is provided. Figure 4 shows the graphs of the pelvis motion, joint angles, and gait phases of two strides. In Figure 4e, the swing and stance phases of the feet are indicated by values 0 and 1, and in Figure 4f, they are shown by unshaded and shaded diagrams, respectively. As shown in the figure, the different motion parameters are correlated to each other. Ref. [25] clearly explained the relationship between the gait phase and different body parts’ displacement as well as orientations. According to [25], the vertical displacement of the sacrum, trunk, and head is equal with each segment and follows a double sinusoidal path per stride. This is evident from the velocity (which is directly proportional to displacement) $v_{z}$ and foot phase p graphs of Figure 4, showing two cycles of velocity per stride. In other words, there are two cycles of downward and upward displacements in each stride. In terms of progressional displacement, the axial segments (upper body including the pelvis) measured during treadmill walking exhibit a relationship to gait velocity that follows a double sinusoidal curve per stride. They discovered that during the first third of each step cycle, the axial segments advance faster than the mean gait velocity. They defined the step cycle as starting 10% of the step cycle before the toe-off. This is illustrated in Figure 4c, where a rapid increase in $v_{x}$ is observed around the toe-off event. This periodic behavior demonstrates the close interrelation among different types of motion parameters. However, ref. [25] only demonstrated the temporal correlation using signal waveforms. As discussed by [26], walking speed influences the cadence, step lengths, and joint rotation ranges. Therefore, walking speed, in addition to gait phase, is a crucial parameter to fully represent this straight walking motion. Thus, the gait phase and walking speed (mean gait speed) were selected as the key motion parameters.

The first phase of the developed framework, motion parameterization, is performed using the encoder network. The network estimates the pelvic velocity and gait phase from the inertial data acquired by the IMU sensor attached to the pelvis. Time- and context-sensitive networks such as LSTM or Bi-LSTM are commonly used for velocity estimation [23,24]. Given that velocity involves integrating acceleration over time, a Bi-LSTM network is implemented for this purpose. Therefore, the network input comprises a history of the previous nine inertial data frames along with the current frame {$a_{t-9},\ldots a_{t}\cup \omega_{t-9},\ldots \omega_{t}$}. This configuration results in the input data having a shape of 10 × 6. The output quantities are the current velocity $v_{t}$ and phase $p_{t}$. For simplicity, only the velocities for straight walking ($v_{x},v_{z}$) were considered, computed from the pelvic position data recorded every 0.01 s by the motion capture system. These velocities are regarded as the ground truth while the phase ground truth is taken from the insole data of the feet. The network comprises 512 LSTM units followed by three dense layers of 128 and 64 units. Lastly, a dense layer with 2 units is added for velocity, and a dense layer with 1 unit followed by a lambda layer, which clips output into the range from 0 to 1, is added for the phase. For training, the dataset was split into training, validation, and testing datasets in the ratios of 75%, 10%, and 15%, respectively.

### 3.2. Motion Synthesis

This part involves a simple deep learning network, referred to as a decoder network, which receives motion parameters from either the encoder network (mode-I) or the Fourier series signal generator (mode-II). The network has a simple structure, which consists of four dense layers with 256, 128, 64, and 6 units, progressing from the input to the output side, respectively. To prevent overfitting, a dropout layer with a ratio of 0.3 was introduced between the second and third layers. This feedforward network translates input motion parameters into corresponding leg joint angles and position changes of the pelvis segment. The position change is the difference between the current and previous positions. Like the encoder network, the input consists of a history of 10 motion frames. As a result, the input shape is 10 × 3 and the output shape is 6 × 1, comprising 4 joint angles and 2 sagittal plane position changes of the pelvis. It is important to note that the ground truth data for joint angles and position change values were directly obtained from the optical motion capture system.

Another interactive method to generate the two motion parameters $v_{t}$ and $p_{t}$ was employed using Fourier series from high-level user inputs. A Fourier series represents a periodic function as an infinite sum of trigonometric functions [27]. Assuming all these signals are periodic, a Fourier series can be utilized to reconstruct the pelvis velocity signals. The Fourier series is formulated as follows [27]:

(1) $$f\left(t\right)=A_{0}+\sum_{n=1}^{6}\left(A_{n}\cos\left(\frac{2\pi nt}{T^{{}^{\prime }}}\right)+B_{n}sin(\frac{2\pi nt}{T{}^{\prime }})\right),\;\mathrm{where}\;n\geq 1\;A_{0}=\frac{1}{T^{{}^{\prime }}}\int_{-\frac{T^{{}^{\prime }}}{2}}^{\frac{T^{{}^{\prime }}}{2}}f\left(t\right)dt\;A_{n}=\frac{2}{T^{{}^{\prime }}}\int_{-\frac{T^{{}^{\prime }}}{2}}^{\frac{T^{{}^{\prime }}}{2}}f\left(t\right)cos(\frac{2\pi nt}{T{}^{\prime }})dt,\;B_{n}=\frac{2}{T^{{}^{\prime }}}\int_{-\frac{T^{{}^{\prime }}}{2}}^{\frac{T^{{}^{\prime }}}{2}}f\left(t\right)sin(\frac{2\pi nt}{T{}^{\prime }})dt$$

Here, $A_{0}$ represents the walking speed while $T{}^{\prime }$, which is half of T, denotes the period of the velocity.

**Figure 5:**
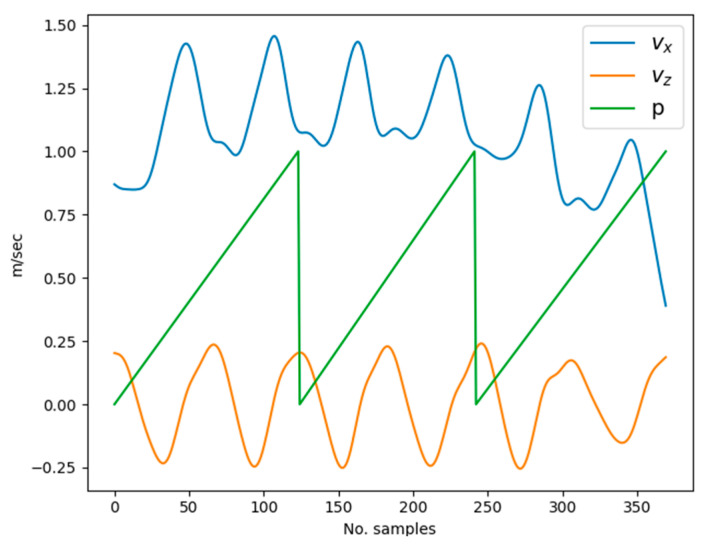
Relation among gait phase (p) and pelvis horizontal ($v_{x}$) and vertical velocities ($v_{y}$).

**Figure 6:**
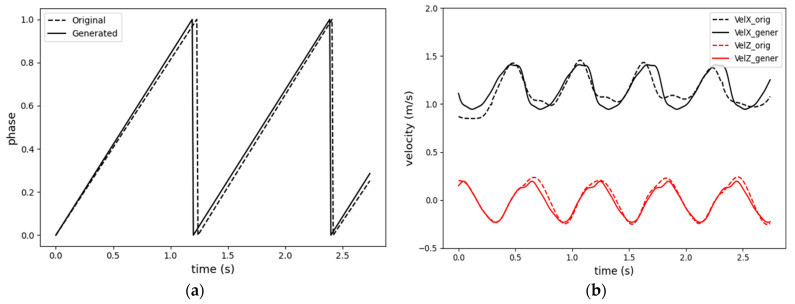
Comparison of the original motion data (dashed line) and Fourier-series-generated data (solid line). (**a**) Generated gait phase; (**b**) vertical and horizontal velocity generation.

As discussed in the previous Section 3.1, pelvis motion is closely correlated with the gait phase. During level walking, the pelvis follows a sinusoidal curve along both the vertical and horizontal (anterior/posterior) axes [25,28]. This cyclic characteristic is depicted in Figure 5, showing the pelvis velocity signals complete two cycles per stride (one period of the gait phase). Since only one subject participated in the data collection, the lower limb body measurements are constant. Given constant leg lengths, straight walking can be represented by the walking speed and gait phases of the feet. Therefore, given the gait period, T, velocity signals can be reconstructed using Fourier series, and the phase signals can be generated using a sawtooth wave generator at T period. Additionally, by varying only T, it is possible to generate different walking motion patterns at a constant velocity. If the velocity is increased while keeping the T constant, the additional velocity must be compensated by larger strides. For instance, the results of a Fourier-series-based reconstruction of original motion capture data with a T of 1.2 s are presented in Figure 6 with original signals shown by dashed lines while the Fourier-series-generated signals are depicted in solid lines. The walking speed command u is adjusted by altering the parameter $A_{0}$. Here, u corresponds to the offset of the forward velocity component from the zero level.

### 3.3. Motion Control

**Figure 7:**
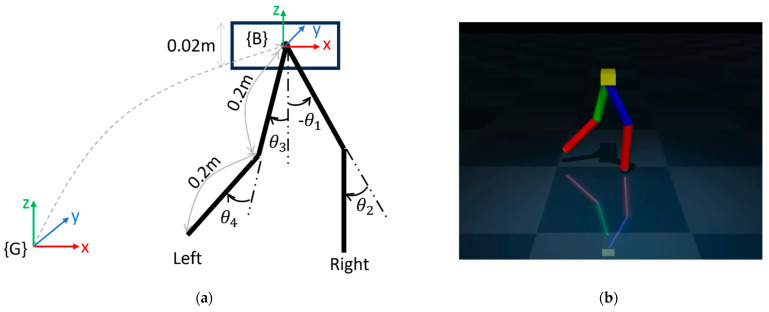
(**a**) Schematics of the biped robot model. {G} and {B} are global and body coordinates, respectively. The axis of rotation for all the joint angles is +*y*-axis. (**b**) Robot model in MuJoCo.

This part of the framework employs a tracking controller to transfer the motion synthesized by the decoder network to the biped robot model. The robot model employed in this study is shown in Figure 7. Each of the four links is modeled as a cylinder, having a radius of 0.02 m, length of 0.2 m, and mass of 0.25 kg. The base is designed as a rectangular box with 0.02 m height, 0.075 m width, 0.03 m depth, and a mass of 0.75 kg. This model was made smaller than the subject’s body dimensions only to reflect the difference between human and robot physical models. Thus, the total mass of the model is 1.75 kg. To simplify the model, the base link has only two degrees of freedom (DOF): translation along x- and z-axes. Therefore, the system has six DOFs in total, with two attributed to the base and four to the leg joints. The six outputs from the decoder network correspond to these six DOF quantities. Hence, a joint-space-based control system was implemented.

The equation of motion of legged systems can be written as [29]

(2) $$q=\left[\begin{array}{c} \begin{array}{c} x\\ z \end{array}\\ \theta_{1}\\ \theta_{2}\\ \theta_{3}\\ \theta_{4} \end{array}\right]=\left[\begin{array}{c} r_{b}\\ q_{j} \end{array}\right]$$

(3) $$M\left(q\right)\ddot{q}+b\left(q,\dot{q}\right)+g\left(q\right)+J_{C}^{T}F_{c}=S^{T}\tau$$

where $q,\dot{q},\ddot{q}\in R^{n_{q}}$ denote the generalized coordinates, velocities, and accelerations, respectively; $M(q)\in R^{n_{q}\times n_{q}}$ is the orthogonal mass matrix; $b\left(q,\dot{q}\right)\in R^{n_{q}}$ represents the Coriolis and centrifugal terms; $g\left(q\right)\in R^{n_{q}}$ denotes the gravitational terms. $F_{c}\in R^{{2n}_{c}}$ is the translational contact force the robot exerts on the environment. This force is mapped to the joint space torques through the contact Jacobian, $J_{c}\in R^{{2n}_{c}\times n_{q}}$ (only translational part). For multiple contacts, $J_{c}={{[J}_{c_{1}},J_{c_{2}},\ldots J_{c_{n_{c}}}]}^{T}$. Lastly, this model has an unactuated base; hence, the selection matrix, $S=[0_{n_{\tau }\times \left(n_{q}-n_{\tau }\right)}I_{n_{\tau }\times n_{\tau }}]$, selects the actuated joints from the generalized torques, $\tau \in R^{n_{\tau }}$. $n_{q},n_{c}$, and $n_{\tau }$ denote the number of generalized coordinate components, the number of contacts, and the number of actuated joints (=4), respectively.

To simplify the complexities of contact forces, contacts are treated as kinematic constraints. Given the contact position of point C on the robot is $r_{c}$ and point C remains static, its motion is constrained as expressed in the following equation.

(4) $$r_{c}=constant;{\;\dot{r}}_{c}=J_{c}\dot{q}=0;\;{\ddot{r}}_{c}=J_{c}\ddot{q}+{\dot{J}}_{c}\dot{q}=0$$

To find an inverse dynamics control method satisfying the constraint in Equation (4), [30] suggested the use of a linear map (null-space matrix), $N_{c}$. This maps the solution manifold of the inverse dynamics into a constraint-consistent solution manifold. The dynamics of the system are rewritten as

(5) $$N_{c}^{T}(M\ddot{q}+b+g)=N_{c}^{T}S^{T}\tau$$

From Equations (3) and (4), the contact force can be written as

(6) $$F_{c}={{(J}_{c}M^{-1}J_{c}^{T})}^{-1}(J_{c}M^{-1}\left(S^{T}\tau -b-g\right)+{\dot{J}}_{c}\dot{q})$$

By substituting Equation (6) into the dynamics Equation (3) and comparing the results with (5), we obtain a dynamically consistent support null-space matrix, $N_{c}$, as

(7) $$N_{c}=I-{{{M^{-1}J}_{c}^{T}(J}_{c}M^{-1}J_{c}^{T})}^{-1}J_{c}$$

With the desired generalized acceleration, ${\ddot{q}}_{des}$, the joint torque can be computed from Equation (5), the dynamic equation of the constraint-consistent equation, rearranged as

(8) $$\tau ={{{(N}_{c}^{T}S^{T})}^{+}N}_{c}^{T}(M{\ddot{q}}_{des}+b+g)$$

**Figure 8:**
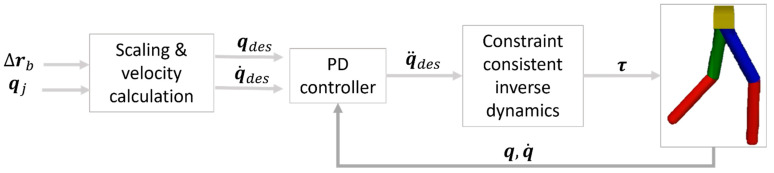
Motion tracking controller.

The superscript ‘+’ denotes the pseudo-inverse of a matrix. Since the decoder network yields $q_{des}$, the desired velocities, ${\dot{q}}_{des}$, are computed from $q_{des}$ with the 0.01 s sampling time. Given the inverse dynamics of the constraint-consistent equation in (8), the ${\ddot{q}}_{des}$ is formulated as a mass-spring model, PD (proportional and derivative) of the position and the velocity terms as shown in (9).

(9) $${\ddot{q}}_{des}=K_{p}\left(q_{des}-q\right)+K_{d}\left({\dot{q}}_{des}-\dot{q}\right)$$

where $K_{p}\in R^{n_{q\times }n_{q}}$ and $K_{d}\in R^{n_{q\times }n_{q}}$ are positive-definite diagonal matrices of proportional and derivative gains of (9). These gain values are tuned manually by checking the simulation of the robot. The robot simulation was implemented using MuJoCo, an open-source physics engine developed by Google’s DeepMind. An overview of the tracking controller can be seen in Figure 8. In this model, first the $\Delta r_{b}$ is scaled down according to the leg length ratio between the human subject and the robot. However, perfect scaling down for the position quantities may not be achieved. Hence, the controller was designed in such a way that it prioritizes tracking the joint angle while fulfilling the contact constraint. This scaling only affects the distance covered by the robot and the human subject.

## 4. Results and Discussion

### 4.1. Results

This section will detail the results of the motion parameterization (encoder), motion synthesis (decoder), and the constraint-consistent controller. All programs for this system were developed using Python programming language and TensorFlow machine learning library on a Dell XPS 15 computer, equipped with NVIDIA GeForce RTX 3050 TI. Additionally, the MuJoCo physics engine was utilized for robot simulation.

**Figure 9:**
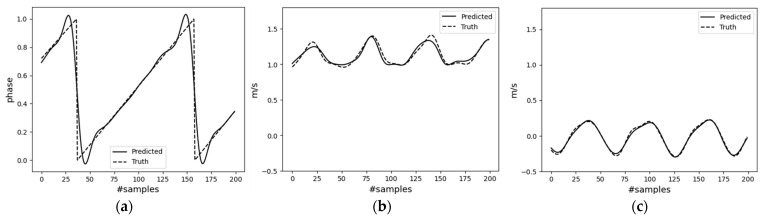
Results of the Bi-LSTM (encoder) network. (**a**) Gait phase; (**b**) horizontal pelvis velocity; (**c**) vertical pelvis velocity.

First, the encoder model results are discussed. The encoder, a Bi-LSTM network, estimates the pelvis’s vertical and horizontal velocities, along with the gait phase. The model underwent training with 100 epochs, a batch size of 32, and an exponentially decaying learning rate starting at 0.0005. Figure 9 shows the results of the trained model when evaluated with the unseen testing dataset. The MAE (mean absolute error) for the three output quantities—namely the pelvis horizontal (forward), vertical, and gait phase—is 0.052 m/s, 0.020 m/s, and 0.039, respectively.

Next, the results of the decoder network will be discussed. Firstly, the decoder network was trained using raw pelvis velocity and phase data from the optical motion capture and the insole system. Once trained with the actual motion data, this trained model was used for later inference tasks. The training hyperparameters of this network are the same as the encoder network.

**Figure 10:**
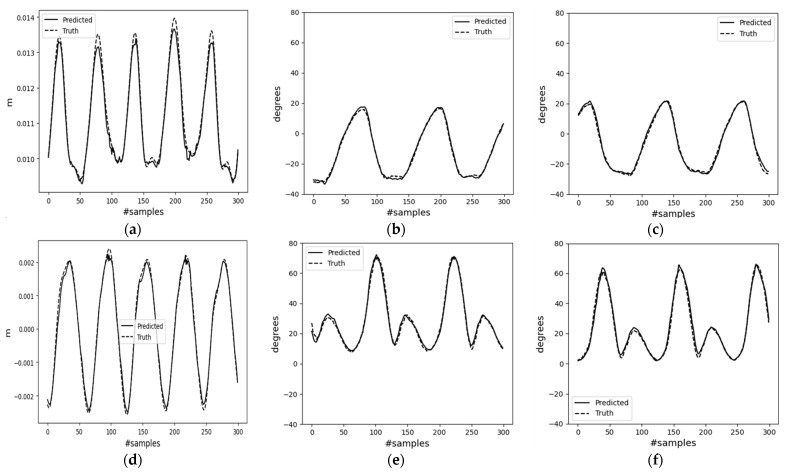
Results of the feedforward (decoder) network for case-1, input data come from the collected motion data, not the encoder, mode-I. (**a**) Position change along the horizontal axis; (**b**) right leg hip joint angle; (**c**) left leg hip joint angle; (**d**) position change along the vertical axis; (**e**) right leg knee joint angle; (**f**) left leg knee joint angle.

**Figure 11:**
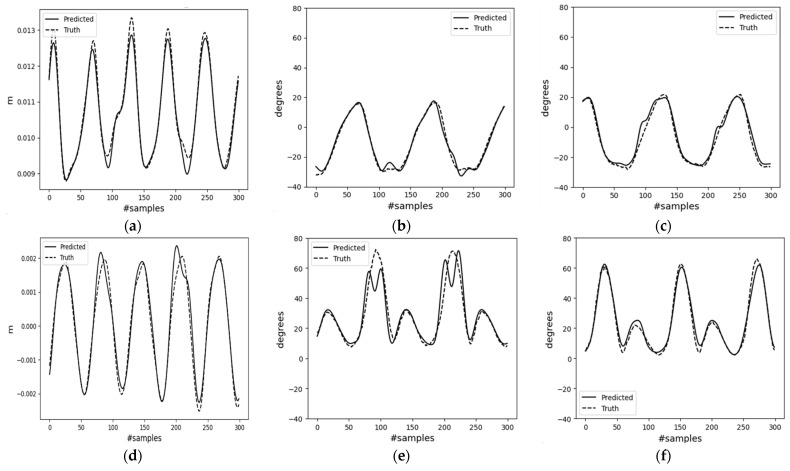
Results of the feedforward (decoder) network for case-2, input data come from the encoder, mode-I. (**a**) Position change along horizontal axis; (**b**) right leg hip joint angle; (**c**) left leg hip joint angle; (**d**) position change along the vertical axis; (**e**) right leg knee joint angle; (**f**) left leg knee joint angle.

During inference, the decoder can take input from different types of data. These include velocity and phase data directly from the testing dataset (case-1); encoder outputs of the testing inertial dataset (case-2) of mode-I; and user-command-generated signals of mode-II. Results for mode-I cases can be found in Figure 10 and Figure 11. Additionally, the MAE results for the trained decoder model in both cases are presented in Table 1. From Table 1, the performance in case-2 falls short compared to that in case-1. This discrepancy may arise from error propagation from the encoder network to the decoder network. The table shows that for case-1, the decoder model achieved a minimum MAE value of 1.35° and a maximum MAE value of 1.75° in joint angle estimation. This suggests that complementing pelvis (base) velocity with precise gait phase values aids in understanding the lower body’s kinematics. In the context of the biped robot, joint rotations are estimated based on the base velocity (position) and phase data of both feet (end effectors). Therefore, changing these parameters allows for the generation of various lower-body motion types. In case-2, the model appears to struggle with approximating the right knee’s maximum flexion. Additionally, one factor in the error propagation from the encoder to the decoder could be selecting the best-trained model based solely on its lowest validation accuracy, without considering the accuracies of individual output variables. Generally, the results of deep-learning methods are difficult to interpret.

**Figure 12:**
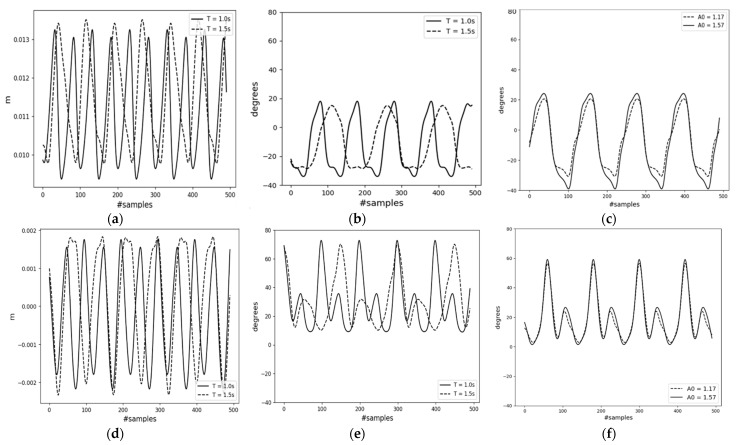
Decoder network results for user commands. (**a**) Position change along the horizontal axis ($A_{0}$ = 1.2); (**b**) right leg hip joint angle ($A_{0}$ = 1.2); (**c**) left leg hip joint angle (T = 1.2); (**d**) position change along the vertical axis ($A_{0}$ = 1.2); (**e**) right leg knee joint angle ($A_{0}$ = 1.2); (**f**) left leg knee joint angle (T = 1.2).

Figure 12 shows a segment of the trained decoder model’s results for mode-II, which used user-generated input data. The first two columns in the figure correspond to gait periods of 1.0 s and 1.5 s, respectively. They are presented in the same figure for easier comparison. These graphs clearly depict the frequency difference, where T = 1.5 s has gait compared to T = 1.0 s. The walking pace parameter $A_{0}$ was kept constant. The last column of Figure 12c,f illustrates the results from input data generated with varying $A_{0}$ while keeping T constant. Figure 12c reveals that faster walking paces are compensated by larger hip joint angles for higher speed, $A_{0}$ = 1.57.

**Figure 13:**
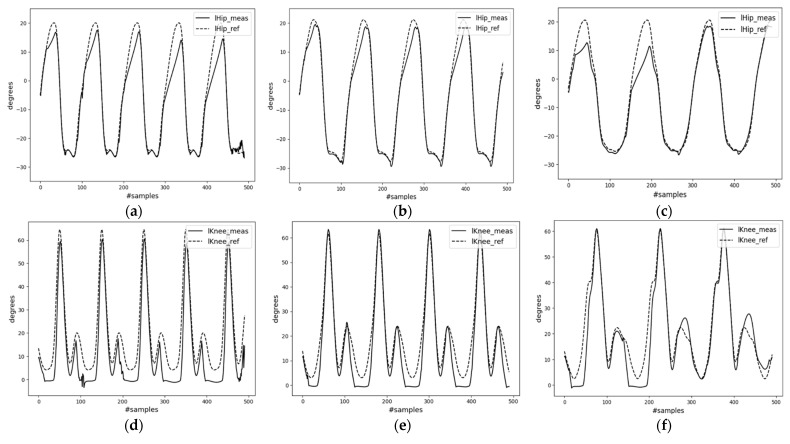

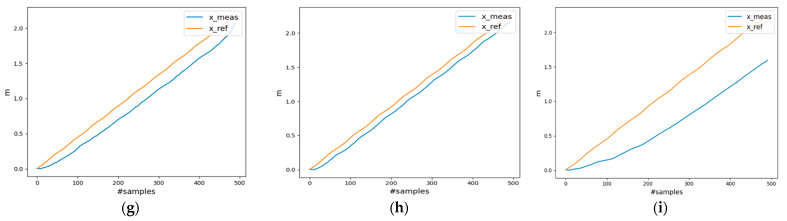
Tracking controller results. lHip_meas = left hip joint measured angle; lHip_ref = left hip joint reference angle; lKnee_meas = left knee joint measured angle; lKnee_ref = left knee joint reference angle; x_meas = measured forward displacement; x_ref = reference forward displacement. Reference data of (**a**,**d**,**g**) are from user command of T = 1.0 s; (**b**,**e**,**h**) are from the encoder output of the testing dataset; (**c**,**f**,**i**) are from user command of T = 1.5 s.

**Figure 14:**
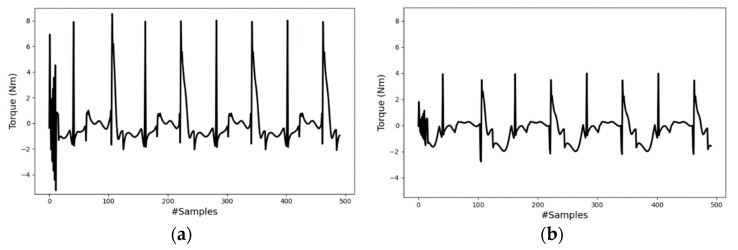
Sample of the computed torques of (**a**) the hip and (**b**) knee joints (left leg).

Figure 13, lastly, presents the results of the tracking controller. In the figure, the solid line represents measured values from the robot simulator, and the dashed line depicts the reference motions obtained from the decoder network. Figure 13b,e,h were derived from the encoder outputs of the testing inertial data (mode-I), whereas the others were obtained using user commands (mode-II). Since covering the walking distance of the indoor area takes less than 5 s, the data were extended using the Fourier series model to represent a longer distance while maintaining the same frequency. Due to the differing scales of the robot model and the subject, the pelvis position/velocity reference data were scaled down by the ratio of the subject leg length to the robot’s leg length, approximately 2.5. As this might introduce new errors to the controller, the PD acceleration model’s gain weights for the pelvis translation quantities were set lower. If the robot accurately tracks the joint angles and adheres to the foot contact constraint Equation (4), more or less the robot will follow the reference pelvis motion with some constant error. In general, the errors observed in the graphs could result from less dynamically inconsistent reference motion, initial robot position conditions, and gain selection. However, despite these errors, the tracking performance for the lower-body kinematics, obtained from a single inertial sensor affixed to the waist, was excellent. Table 2 compares the mean absolute errors (MAEs) of the controller results for both modes. The maximum tracking error for the joint angles is 4.12°. For reference, part of the computed torques of the hip and knee joints are presented in Figure 14.

### 4.2. Discussion

The experimental results demonstrate the potential of a single IMU sensor in predicting key human motion parameters and reconstructing human motion from a few parameters using deep learning algorithms. Employing a constraint-consistent inverse dynamics model, a biped robot was controlled to follow generated reference motion data. Overall, the synthesized motion from the single inertial sensor showed promising results, as indicated by the controller’s performance. For motion parameterization, a Bi-LSTM encoder was used to effectively estimate crucial walking motion parameters such as the pelvis velocity and gait phase. The small MAEs of the horizontal and vertical pelvis velocities and gait phase, at 0.052 m/s, 0.020 m/s, and 0.039, respectively, highlight the strong correlation between the waist inertial data and the parameters, velocity and gait phase. Given that the inertial data include acceleration, establishing a connection with the velocity parameter is easier, as velocity is derived from the integral of acceleration. Regarding the gait phase, the foot–ground impact and the swaying motion aided the model in discerning the relationship between waist inertial data and the foot gait phases. Although the encoder’s MAE values are relatively low, these errors could potentially propagate to the subsequent motion synthesis and motion control systems. This is evident from the performance comparison of the decoder model when it takes inputs directly from the encoder’s outputs (case-2) versus actual motion data (case-1). The average MAE values for the joint angles indicate that case-1 (1.56°) achieved approximately 50% better accuracy compared to case-2 (3.2°). This discrepancy demonstrates how errors in the encoder can significantly affect subsequent systems. The results of the decoder model for mode-II, input data, come from user commands, showcasing the model’s ability to generate a variety of motion patterns using only two motion parameters. The capability to adjust parameters such as walking pace and gait period and observe corresponding changes in joint angles underscores the potential of extending the proposed framework to 3D real-world robotic applications. Finally, the tracking controller demonstrated promising motion tracking capability for a synthesized reference motion, with errors less than 5° in the joint angles. However, errors were noted particularly during the full extension of the knee joints. These errors could have arisen from various factors, including initialization, dynamic inconsistency, scaling issues, and gain selection. The impact of the scaling issue is clearly visible in Figure 13i. However, the distance covered by the robot and the input position information are less critical, provided the robot accurately tracks the joint angles and meets the foot contact constraint, as is shown in Figure 13c,f. In the initial phase of the simulation, it is observed that the robot’s measured forward displacement lags behind the reference. This led the robot to move its legs hastily without fully bending the joint angles. This phenomenon is illustrated in the first half of the graphs in Figure 13c,f,i.

Despite the achieved results, the study has its limitations. One such limitation is that the developed system cannot be generalized to all walking patterns, as only straight-level walking was investigated. Consequently, different walking types involving momentary stopping, traversing uneven terrain, turning, and transitioning between walking and standing were not considered in this study. As a result, the system does not generalize across all forms of walking patterns. The limitation of Fourier-series-based signal generation lies in its applicability only to cyclic motions. Fourier series are ideal for steady-state periodic signals but may not handle transient behaviors well. Therefore, for non-cyclical or irregular motions, particularly in dynamic human activities beyond walking, an alternative parameter-to-motion mapping algorithm is required. Another limitation is the inherent lack of interpretability in deep learning algorithms. This may pose difficulty in fully understanding the system, as neural networks are often considered black boxes. Moreover, the study did not investigate other control algorithms and different robot model parameters. Hence, the controller results could potentially be improved through the introduction of more advanced joint space control algorithms. Lastly, since the robot model adopted is 2D, it does not fully correspond to the full 3D human motion data as it ignored four DOFs of the base link including the 3D rotation and sideways translation motions.

## 5. Conclusions and Future Works

This study presented a comprehensive approach to motion parameterization, motion synthesis, and motion control, based on deep learning and using a single inertial sensor attached to the subject’s waist. These three components were implemented sequentially. The motion parameterization phase adopted a Bi-LSTM encoder network. The encoder accurately predicted key parameters such as pelvis velocities and gait phase from the waist inertial data achieving an MAE of 0.052 m/s, 0.020 m/s, and 0.039, respectively. The motion synthesizer, a feedforward decoder network, utilizes these predictions to provide insights into lower-body kinematics. By modifying these parameters, the system generated various walking patterns for the biped robot. The generated motions were inputted as reference to the biped motion controller, which tracked these motions with an MAE of less than 5°. Furthermore, the motion tracking controller adapted to various synthesized motions, underscoring the potential of extending this approach to real-world scenarios.

While the results are promising, there is potential for improvements across all three stages of the framework. These improvements could be in motion measurement, the choice and training of deep learning algorithms, and the integration of robust modern control algorithms. Future work will focus on enhancing the encoder’s accuracy by incorporating signal processing methods to ensure the output gait phase closely resembles the actual gait phase, and the scaling issues will be investigated. Additionally, the study will be extended to include a variety of 3D walking motions across different terrains. Therefore, developing a robust 3D tracking control system is part of the study’s future work. Therefore, this study lays the groundwork for extending our work to include more dynamic motions, motion transitions, and diverse walking environments, thereby broadening the range of motion types.

## Figures and Tables

**Table 1:** MAE of the decoder network when tested with case-1 and case-2 datasets. posX and posZ denote the position change of the pelvis part.

Cases	posX (mm)	posZ (mm)	Hip Right (°)	Knee Right (°)	Hip Left (°)	Knee Left (°)
Case-1	0.12	0.14	1.35	1.73	1.42	1.75
Case-2	0.16	0.25	2.44	5.16	2.32	2.82

**Table 2:** MAE comparison of the motion tracking controller results for mode-I and mode-II (*T* = 1.0). The X and Z terms denote the distance travelled along x and z axes.

Mode	X (m)	Z (m)	Hip Right (°)	Knee Right(°)	Hip Left (°)	Knee Left (°)
Mode-I	0.11	0.05	2.01	2.88	2.22	3.11
Mode-II	0.18	0.03	4.33	2.21	2.78	4.12

## Data Availability

Partial resources of this research can be found at https://github.com/tsgtdss583/motionGene_robotControl (accessed on 5 October 2023).
